# Oxidized LDL enhances Gq signaling and aldosterone production by angiotensin II via the AT1-LOX-1 receptor complex in adrenal cells

**DOI:** 10.1038/s41440-025-02261-5

**Published:** 2025-06-18

**Authors:** Jittoku Ihara, Yibin Huang, Yoichi Takami, Yu Guo, Toshimasa Takahashi, Akemi Kakino, Yoichi Nozato, Cheng Wang, Ziwei Wang, Weidong Liu, Nanxiang Yin, Ryoichi Ohara, Akitoshi Hara, Hikari Takeshita, Hiromi Rakugi, Tatsuya Sawamura, Koichi Yamamoto

**Affiliations:** 1 Department of Geriatric and General Medicine, Osaka University Graduate School of Medicine, Suita, Osaka Japan; 2 Center for Pulmonary and Vascular Biology, Department of Pediatrics, University of Texas Southwestern Medical Center, Dallas, TX USA; 3 Department of Medicine, University of Toronto, Toronto, ON Canada; 4 Department of Molecular Pathophysiology, Shinshu University Graduate School of Medicine, Matsumoto, Nagano Japan

**Keywords:** Adrenal cells, Aldosterone, Angiotensin II, AT1-LOX-1 receptor complex, Oxidized LDL

## Abstract

We previously reported that oxidized low-density lipoprotein (oxLDL) activates the angiotensin II (AII) type 1 receptor (AT1) through the lectin-like oxLDL receptor (LOX-1)-AT1 complex. While oxLDL alone does not activate G protein αq (Gq), its simultaneous binding with AII alters AT1 structure, enhancing Gq activation. We investigated this interaction’s effect on aldosterone production in adrenal glands. Human adrenal cells (H295R) were treated with vehicle, oxLDL, AII, or a combination of oxLDL and AII. Gq signaling was assessed using inositol monophosphate (IP1) assays, Ca influx using Fura2, and aldosterone synthesis gene expression using qRT-PCR. Wild-type (WT) and LOX-1 knockout (KO) mice were fed a normal diet (ND) or high-fat diet (HFD) in vivo to elevate oxLDL levels, followed by subcutaneous AII or saline infusion for 4 weeks (long-term) or 3 days (short-term). In H295R cells, oxLDL alone did not induce IP1 production; however, AII-induced IP1 and Ca influx were enhanced by oxLDL. These effects were abolished by LOX-1 siRNA. Co-administration of oxLDL and AII upregulated aldosterone synthesis genes, such as *CYP11B2*; this effect was suppressed by a Gq inhibitor. In vivo, long-term AII and HFD administration increased adrenal CYP11B2 expression but not serum aldosterone levels. Conversely, short-term AII and oxLDL administration elevated serum aldosterone levels, but not CYP11B2 expression. These effects were absent in LOX-1 KO mice. Blood pressure was unaffected by HFD or oxLDL in both models. In conclusion, OxLDL enhances AII-induced aldosterone production in adrenal glands through LOX-1-AT1 interaction, although its impact on blood pressure regulation remains unclear.

**Figure:**
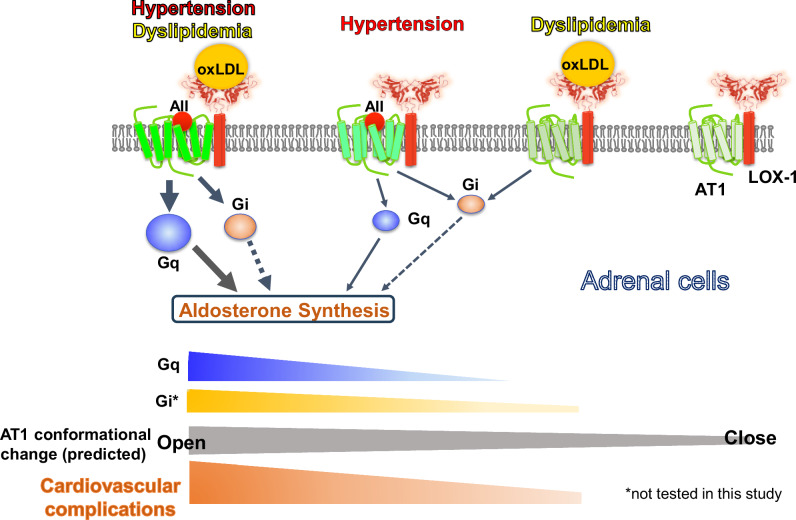
Oxidized LDL enhances Gq signaling and aldosterone production by angiotensin II via the AT1-LOX-1 receptor complex in adrenal cells. LOX-1 amplifies angiotensin II-induced aldosterone synthesis by modulating AT1 receptor signaling in adrenal glands. Co-stimulation with oxLDL enhances Gq activation via LOX-1-AT1 interaction, potentially through AT1 unique conformational changes. These findings underscore the interplay between dyslipidemia and RAAS in promoting hypertension and cardiovascular diseases

Oxidized LDL enhances Gq signaling and aldosterone production by angiotensin II via the AT1-LOX-1 receptor complex in adrenal cells. LOX-1 amplifies angiotensin II-induced aldosterone synthesis by modulating AT1 receptor signaling in adrenal glands. Co-stimulation with oxLDL enhances Gq activation via LOX-1-AT1 interaction, potentially through AT1 unique conformational changes. These findings underscore the interplay between dyslipidemia and RAAS in promoting hypertension and cardiovascular diseases

## Abstract (web summary)

Oxidized LDL enhances Gq signaling and aldosterone production by angiotensin II via the AT1-LOX-1 receptor complex in adrenal cells. LOX-1 amplifies angiotensin II-induced aldosterone synthesis by modulating AT1 receptor signaling in adrenal glands. Co-stimulation with oxLDL enhances Gq activation via LOX-1-AT1 interaction, potentially through AT1 unique conformational changes. These findings underscore the interplay between dyslipidemia and RAAS in promoting hypertension and cardiovascular diseases

## Introduction

Dyslipidemia and hypertension are major risk factors for cardiovascular diseases and are frequently associated with enhanced activation of the renin-angiotensin-aldosterone system (RAAS) [1]. The RAAS plays a central role in regulating blood pressure, electrolyte balance, and fluid homeostasis, with angiotensin II (AII) serving as a key effector peptide that exerts its effects primarily through the angiotensin II type 1 receptor (AT1) [2]. Activation of AT1 triggers a cascade of signaling pathways, including the activation of G protein αq (Gq), which subsequently promotes aldosterone production and vasoconstriction [3].

Numerous studies have demonstrated that statins, which are widely prescribed for dyslipidemia, also exert a modest blood pressure-lowering effect [4], suggesting a potential causal relationship between dyslipidemia and hypertension. Oxidized low-density lipoprotein (oxLDL), which is elevated in dyslipidemia, plays a central role in the development of atherosclerosis, and exerts various pathological effects on the cardiovascular system [5]. Our previous studies have revealed that oxLDL can activate AT1 through its interaction with the lectin-like oxLDL receptor (LOX-1) on cellular membranes [6–8]. However, the effect of this interaction on aldosterone production in adrenal cells and its contribution to the pathophysiology of hypertension and cardiovascular diseases associated with dyslipidemia remain unclear.

Recently, we reported that simultaneous binding of oxLDL and AII to their respective receptors, LOX-1 and AT1, enhanced Gq activation in CHO (Chinese hamster ovary) cells engineered to express both the receptors [8]. This enhanced Gq activation increased inositol phosphate 1 (IP1) production and calcium influx, indicating a synergistic interaction between oxLDL and AII within the AT1-LOX-1 receptor complex. Notably, a study reported that AT1 exhibits distinct modes and degrees of G protein activation, depending on ligand-induced conformational changes [9]. Thus, our findings suggested that the simultaneous interaction between oxLDL and AII induces a unique conformational change in AT1, which is distinct from that induced by either ligand alone.

In vivo studies in mice fed a high-fat diet (HFD) to elevate oxLDL levels, combined with AII infusion, demonstrated exacerbated renal injury and dysfunction compared to mice treated with AII alone. These results suggest a novel mechanism underlying renal injury and dysfunction in chronic kidney disease (CKD) driven by dyslipidemia and hypertension. Interestingly, this synergistic enhancement of Gq signaling was observed in renal epithelial and fibroblast cells but not in vascular endothelial cells, vascular smooth muscle cells, or macrophages, highlighting cell-specific differences in response.

In the present study, we investigated the effect of oxLDL on AII-induced aldosterone production in adrenocortical cells via the AT1-LOX-1 receptor complex, particularly focusing on the potential role of Gq signaling in this process. We hypothesized that simultaneous binding of oxLDL and AII to their respective receptors would amplify Gq activation, leading to increased aldosterone production in adrenal cells. Additionally, we evaluated the relevance of this phenomenon in vivo by examining whether the interaction between oxLDL and AII enhanced aldosterone production and subsequently influenced blood pressure in mice.

## Methods

### Cell culture and materials

The human adrenal gland carcinoma cell line H295R [10] (Cosmo Bio, Japan) was cultured in DMEM (Wako, Japan) supplemented with 5% fetal bovine serum (FBS) and 1% penicillin-streptomycin. Human aortic smooth muscle cells (HASMCs) were cultured in SmBM medium supplemented with SmGM- 2 SingleQuots (LONZA, Basel, Switherlad). The cells were maintained at 37 °C under an atmosphere of 5% CO₂ and 95% air.

### Small interfering RNA

H295R cells were seeded at 50% confluence on the day of transfection. Silencer Select small interfering RNAs (siRNAs) targeting *LOX-1* and *AT1a* (Thermo Fisher Scientific, MA, USA) were transfected into the cells using Lipofectamine RNAiMAX (Thermo Fisher Scientific) in serum- and antibiotic-free medium, following the manufacturer’s protocol.

### Preparation of oxLDL

Human plasma LDL (density: 1.019–1.063 g/mL), isolated using sequential ultracentrifugation, was oxidized with 20 μM CuSO₄ in phosphate buffered saline (PBS) at 37 °C for 24 h. The oxidation process was terminated by adding excess EDTA. Oxidation of LDL was assessed using agarose gel electrophoresis to evaluate the migration of oxidized LDL relative to native LDL [6].

### Quantification of cellular IP1 accumulation

Gq-dependent activation of phospholipase C (PLC) was quantified by measuring IP1 levels using the IP-One assay kit (Cisbio, France), as previously described [11]. Briefly, cells were seeded at a density of 80,000 cells/well in 96-well transparent cell culture plates and incubated under serum-free conditions for 24 h. Subsequently, the cells were treated for 1 h with an IP1 stimulation buffer-containing vehicle, oxLDL, AII, and the Gq inhibitor YM-254890 (Fujifilm Wako, Osaka, Japan), as described in the text. After treatment, cell lysates were prepared using Triton X at a final concentration of 1%, and transferred to a 384-well white plate. IP1 levels were measured by incubating the cell lysates with FRET reagents, including a cryptate-labeled anti-IP1 antibody and a d2-labeled IP1 analog.

### Quantitative real-time PCR

Total RNA was purified using the RNeasy Mini Kit (Qiagen, Germantown, MD, USA). Subsequently, 1 µg of RNA was reverse-transcribed into cDNA using the ReverTra Ace qPCR RT Kit (TOYOBO, Osaka, Japan) according to the manufacturer’s protocol. Quantitative real-time PCR was performed using the ViiA7 Real-Time PCR System (Applied Biosystems, Thermo Fisher Scientific). Gene expression levels were analyzed using the ΔΔCt method, with GAPDH serving as the internal reference for normalization. Primer sequences are provided in the Supplemental Table.

### Calcium influx assay

Calcium influx was assessed using Fura-2 AM (Dojindo, Kumamoto, Japan) with slight modifications to the manufacturer’s protocol. Briefly, cells plated in 96-well plates were incubated with 5 μM Fura-2 AM in HEPES-buffered saline (20 mM HEPES, 115 mM NaCl, 5.4 mM KCl, 0.8 mM MgCl_2_, 1.8 mM CaCl_2_, 13.8 mM glucose, and pH 7.4) for 1 h at 37 °C. After incubation, the solution was replaced with the recording medium lacking Fura-2 AM. Next, the cells were treated with oxLDL, AII, or a combination of both at the specified concentrations. Changes in the F340/F380 ratio, an indicator of intracellular calcium concentration, were measured using dual-excitation microfluorometry with a digital image analyzer (Aquacosmos; Hamamatsu Photonics, Hamamatsu, Japan).

### Animal experiments

Male wild-type (WT) mice (C57BL/6 J) and LOX-1 knockout (KO) mice with a C57BL/6 genetic background were used in this study. For long-term interventions, 8-week-old mice were used, whereas 12-week-old mice were used for short-term interventions. LOX-1 KO mice were generated as described previously [12]. All the animals were housed in a temperature-controlled environment (20–22 °C) under a 12-h light/dark cycle.

The mice underwent either short-term or long-term interventions as detailed below:

#### Short-term study

Mice were subcutaneously implanted with osmotic minipumps (Alzet^®^ 2004, Durect Corporation, Cupertino, USA) delivering saline (vehicle), AII, oxLDL, or a combination of AII and oxLDL. The infusion rates were 0.4 γ and 0.25 γ, respectively.

#### Long-term study

Blood samples and adrenal glands were collected from mice according to a method described previously [8]. Briefly, mice were fed either a normal diet (ND; MF, Oriental Yeast, Osaka, Japan) or a HFD without DL-α-tocopherol (CLEA Japan Inc., Tokyo, Japan) that has been shown to increase plasma LOX-1 ligands in ApoE KO mice [13]. AII (0.1 γ) or saline (vehicle) was infused via subcutaneous osmotic minipumps for four weeks.

Figure 4A represents an acute model in which a relatively high dose of AII (0.4 μg/kg/min) was administered for 3 days, along with direct oxLDL infusion, allowing for assessment of rapid aldosterone synthesis and blood pressure responses. In contrast, Fig. 5A represents a chronic model where a lower dose of AII (0.1 μg/kg/min) was continuously administered for four weeks in combination with HFD to increase circulating LOX-1 ligands. This approach aimed to investigate the effects of prolonged exposure to AII under metabolic conditions that promote chronic LOX-1 activation.

All experimental protocols were approved by the Animal Care and Use Committee of Osaka University and conducted in accordance with the NIH guidelines for the Care and Use of Laboratory Animals.

### Blood pressure measurement in mice

Blood pressure (BP) was measured using the tail-cuff method using the BP-98A system (Softron, Japan). Measurements were taken after the mice were properly restrained, and the blood pressure of each animal at each time point was calculated as an average of six readings.

### Tissue preparation

Mice were perfused with cold PBS before excision of adrenal glands and aortas. The adrenal gland and aorta samples were rapidly collected and stored at 4 °C in RNAlater (Thermo Fisher Scientific) to preserve integrity of RNA for its subsequent extraction.

### Statistical analyses

All data are expressed as the mean ± SEM. Comparisons between two groups were performed using Student’s *t*-test, whereas differences among multiple groups were analyzed using one-way ANOVA followed by Tukey’s post hoc test for multiple comparisons.

## Results

### Oxidized LDL potentiates AII-induced IP1 production in a LOX-1-dependent manner in H295R cells

First, we investigated the potentiating effect of oxLDL on AII-stimulated IP1 production, a marker of AT1 receptor-Gq signaling activation, in the adrenocortical cell line H295R that endogenously expresses LOX-1 and AT1 receptors.

**Fig. 1:**
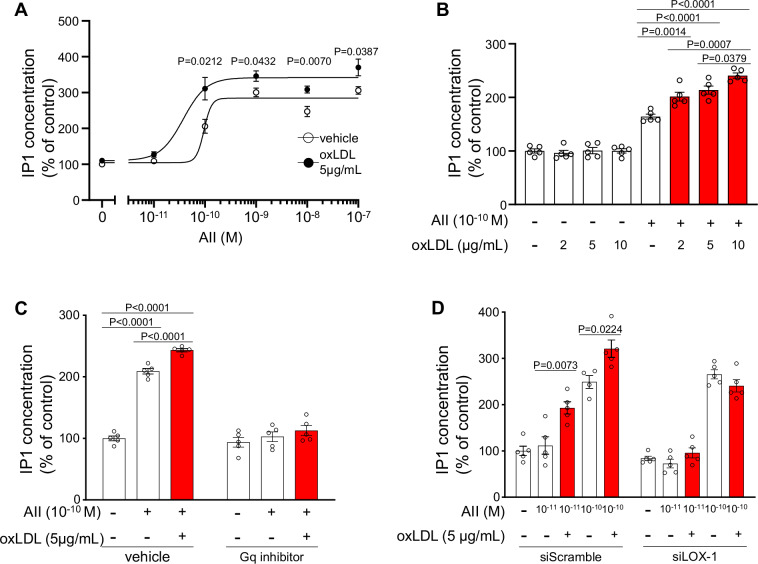
OxLDL potentiates AII-induced IP1 production in a LOX-1-dependent manner in H295R cells. **A** Dose-response curve of AII-induced IP1 production in the presence of varying concentrations of oxLDL in H295R cells. IP1 production reflects the activation of Gq signaling. Cells were treated with oxLDL and AII at the concentrations specified in the figure (n = 5 per oxLDL concentration). **B** IP1 concentration (% of control) in response to vehicle, AII (10^−10^ M), and AII combined with oxLDL at the indicated concentrations in H295R cells. **C** IP1 concentration (% of control) in response to treatment with AII (10^−10^M), oxLDL (5 μg/mL), and their combination, with or without the Gq inhibitor YM-254890, in H295R cells (n = 5 for each group). **D** IP1 concentration (% of control) in response to treatment with AII (10^−10^M or 10^−10^ M) and oxLDL (5 μg/mL), with or without siRNA-mediated knockdown of LOX-1, in H295R cells (n = 5 per group). Data are presented as mean ± SEM. Statistical differences were assessed using Student’s t test (**A**) or one-way ANOVA followed by Tukey’s multiple comparison test (**B–D**)

Figure 1A illustrates the dose-response curve of AII-induced IP1 production, measured as an indicator of Gq activity, in the presence of either vehicle or 5 μg/mL oxLDL in H295R cells. Although oxLDL alone did not induce IP1 production, simultaneous administration of oxLDL significantly enhanced AII-induced IP1 production (Fig. 1A, B). Notably, oxLDL caused a leftward shift in the AII dose-response curve, reducing the half-maximal effective concentration (EC50) by 54% (EC50:9.57 × 10^–11^ M with 0 μg/mL oxLDL; 3.76 × 10^–11^ M with 5 μg/mL oxLDL) (Fig. 1A).

This enhancement of AII-induced IP1 production by oxLDL was abrogated by treatment with the Gq inhibitor YM-254890 (Fig. 1C) or by siRNA-mediated gene silencing of LOX-1 (Fig. 1D).

### OxLDL potentiates AII-induced intracellular calcium influx in H295R cells

**Fig. 2:**
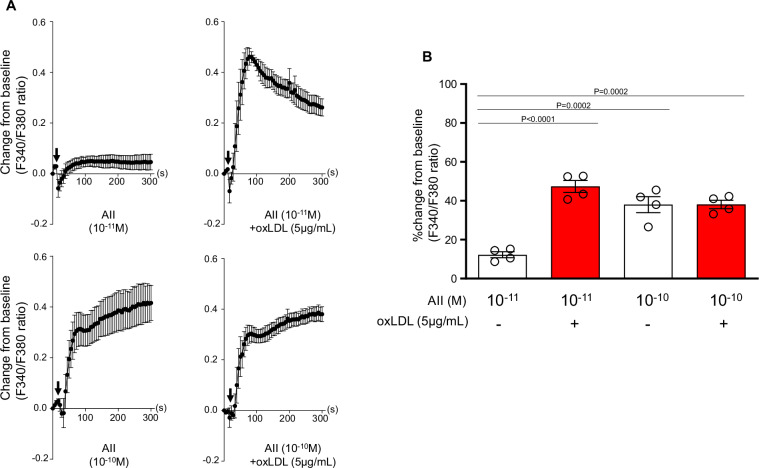
OxLDL potentiates AII-induced intracellular calcium influx in H259R cells. **A** Intracellular calcium concentration in H295R cells was assessed using Fura 2-AM and dual-excitation microfluorometry. Changes in the fluorescence intensity ratio (F340/F380) were used as an index of calcium dynamics. Cells were exposed to AII (10^−10^ or 10^−10^ M) and a combination of oxLDL (5 μg/mL) with AII (10^−10^ or 10^−10^M). Arrows indicate the addition of these agonists during the assay timeline. Real-time calcium flux was monitored using a digital image analyzer (n = 4). **B** Percentage changes from baseline in the F340/F380 emission signal ratio were quantified following treatment with AII (10^−10^ or 10^−10^M) in the presence or absence of oxLDL (5 μg/mL) in H295R cells (n = 4 per group). Data are presented as mean ± SEM. Statistical differences were determined using one-way ANOVA followed by Tukey’s multiple comparison test (**B**)

Intracellular calcium influx is a well-recognized cellular response that is mediated by AII-AT1-Gq activation. Our findings demonstrated that AII at a concentration of 10^−10^ M did not elicit calcium influx in H295R cells when administered alone. However, co-treatment with 5 μg/mL oxLDL significantly induced calcium influx under the same conditions (Fig. 2A, B). Conversely, AII alone at a higher concentration (10^−10^M) triggered calcium influx independently, with no additional enhancement observed upon supplementation with 5 μg/mL oxLDL (Fig. 2A, B).

### Co-treatment of oxLDL with AII enhances cellular response through increased CYP11B2 expression in H259R cells

**Fig. 3:**
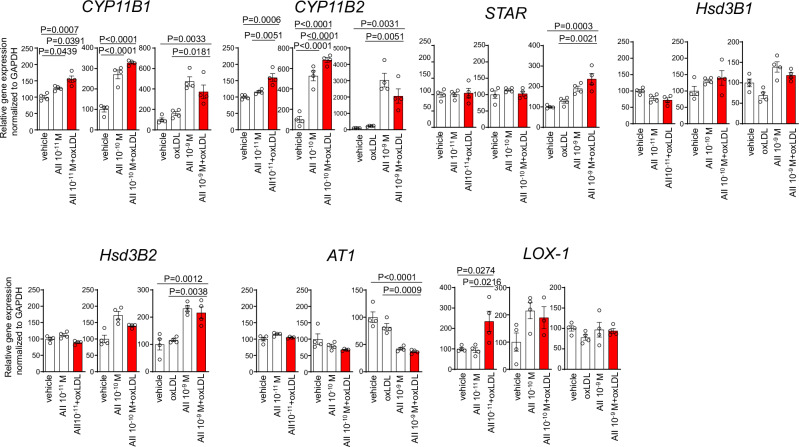
Co-treatment of oxLDL with AII enhanced cellular response via CYP11B2 expression in H259R cells. Expression levels of aldosterone synthesis-related genes, including *CYP11B1*, *CYP11B2*, *STAR*, *Hsd3B1*, *Hsd3B2*, *AT1*, and *LOX-1*, were assessed in H295R cells treated with vehicle, AII (10^−11^, 10^−10^, or 10^−9^ M), or a combination of AII and oxLDL (5 μg/mL). Data are presented as mean ± SEM. Statistical differences were evaluated using one-way ANOVA followed by Tukey’s multiple comparison test

In H259R cells, the expression of the aldosterone synthesis enzyme CYP11B2 and the cortisol synthesis enzyme CYP11B1 was significantly upregulated following co-treatment with AII and 5 μg/mL oxLDL compared to treatment with AII alone, at both low and high doses of AII (Fig. 3). Additionally, LOX-1 expression was significantly upregulated by the combined administration of AII and oxLDL than by AII alone at lower doses of AII but not at higher doses.

### LOX-1-dependent synergistic effect of AII and oxLDL on plasma aldosterone, independent of aldosterone synthesis gene expression, in short-term treatment

**Fig. 4:**
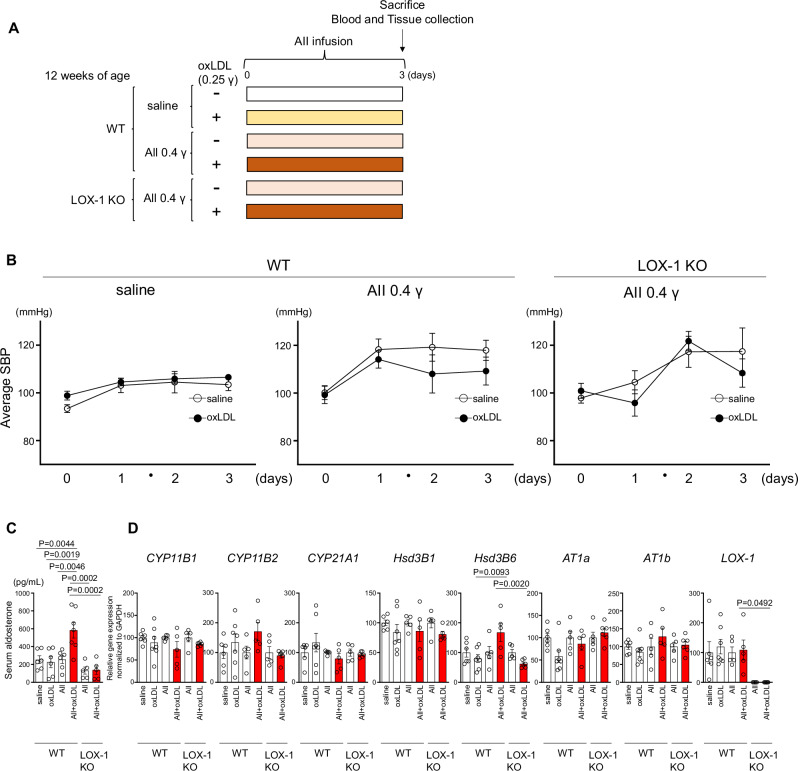
LOX-1-dependent synergistic effect of AII and oxLDL on plasma aldosterone levels, independent of aldosterone synthesis gene expression, in short-term treatment. **A** Schematic protocol of the animal experiments: Twelve-week-old male mice were treated for 3 d with infusions of either saline or 0.4 γ AII in wild-type (WT) mice and 0.4 γ AII in LOX-1 knockout (KO) mice. Vehicle or 0.25 γ oxLDL was simultaneously infused. Reagents were delivered via subcutaneously implanted osmotic pumps. At the end of the infusion period, the animals were sacrificed, and blood samples and adrenal glands were collected for measurement of plasma aldosterone concentration and real-time qPCR analysis to assess the expression of aldosterone synthesis-related genes. **B** Systolic blood pressure trajectory: Serial systolic blood pressure (SBP) measurements obtained using the tail-cuff method are shown for WT and LOX-1 KO mice. The data depict SBP changes corresponding to the administration of saline, 0.4 γ AII to WT mice, and 0.4 γ AII to LOX-1 KO mice, co-treated with vehicle or oxLDL infusion. **C** Plasma aldosterone levels in 12-week-old WT and LOX-1 KO mice after 3 d of treatment, as outlined in Fig. 4A. **D** Real-time qPCR analysis for gene expression of aldosterone synthesis-related molecules (*CYP11B1*, *CYP11B2*, *CYP21A1*, *Hsd3B1*, *Hsd3B6*, *AT1a*, *AT1b*, and *LOX-1*) in adrenal glands harvested from WT and LOX-1 KO mice following 3 d of treatment, as described in (**A**). Data are presented as mean ± SEM. Statistical differences were assessed using Student’s t-test (B) or one-way ANOVA followed by Tukey’s multiple comparison test (**C**, **D**) (n = 5–7 per group)

In this study, we investigated the short- and long-term effects of simultaneous increases in AII and oxLDL levels in vivo. For the short-term analysis, a 3-d infusion of 0.4γ AII combined with oxLDL was administered (Fig. 4A). Short-term treatment with AII (0.4γ) alone induced a modest elevation in BP; however, the addition of oxLDL did not exacerbate increases in BP in either WT or LOX-1 KO mice (Fig. 4B).

Interestingly, the co-administration of AII and oxLDL resulted in a significant increase in plasma aldosterone levels compared to administration of AII alone in WT mice (Fig. 4C). This synergistic effect on plasma aldosterone was absent in the LOX-1 KO mice (Fig. 4D). However, gene expression analysis revealed no significant differences in aldosterone synthesis-related genes, including AT1 and LOX-1 genes, across the treatment groups (Fig. 4D).

### LOX-1-dependent synergistic effect of AII and HFD on aldosterone synthesis gene expression, independent of plasma aldosterone levels, in long-term treatment

**Fig. 5:**
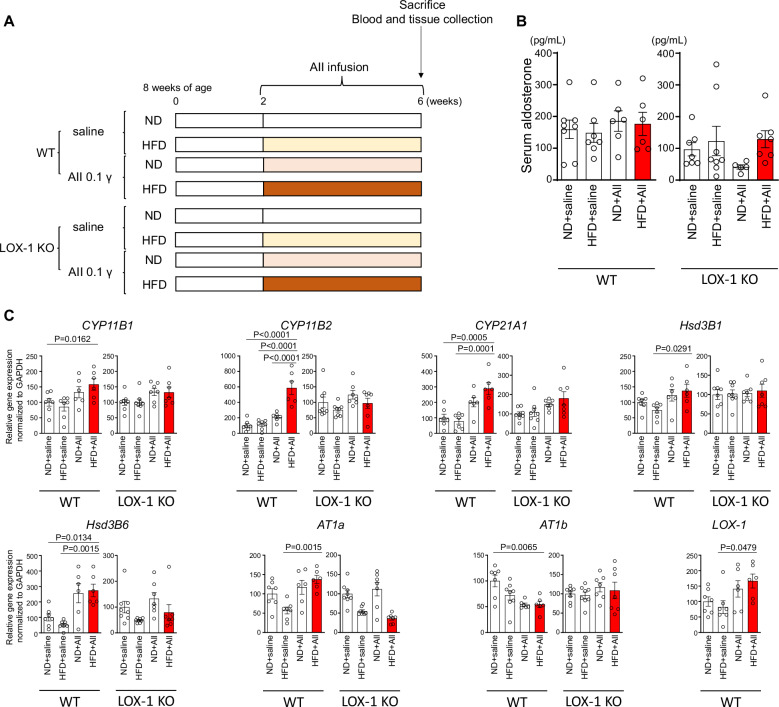
LOX-1-dependent synergistic effect of AII and high fat diet (HFD) on aldosterone synthesis gene expression, independent of plasma aldosterone levels, in long-term treatment. **A** Schematic protocol for the animal experiments: Eight-week-old male WT and LOX-1 KO mice were fed either a normal diet (ND) or HFD for 6 weeks. At 10 weeks of age, the mice received a 4-week infusion of either saline or AII at a subpressor dose of 0.1 γ, delivered via subcutaneously implanted osmotic pumps. At the end of the infusion period, the animals were sacrificed, and blood samples and adrenal glands were collected for measurement of plasma aldosterone concentration and real-time qPCR analysis to evaluate the expression of aldosterone synthesis-related genes. **B** Plasma aldosterone levels in 8-week-old WT and LOX-1 KO mice following the treatment protocol described in Fig. 5A. **C** Real-time qPCR analysis for gene expression of aldosterone synthesis-related molecules (*CYP11B1*, *CYP11B2*, *CYP21A1*, *Hsd3B1*, *Hsd3B2*, *AT1a*, *AT1b*, and *LOX-1*) in the adrenal glands harvested from WT and LOX-1 KO mice after 4 weeks of treatment, as detailed in Fig. 5A. Data are presented as mean ± SEM. Statistical differences were assessed using one-way ANOVA followed by Tukey’s multiple comparison test (B and C) (n = 6–8 per group)

For the long-term study, we utilized a 28-d infusion of 0.1γAII combined with HFD that was known to induce plasma LOX-1 ligands (Fig. 5A), as recently reported [8]. Consistent with our previous findings [8], long-term AII (0.1γ) treatment with or without HFD did not affect BP. Similarly, plasma aldosterone concentrations remained unchanged across all the groups (Fig. 5B).

Notably, the combination of AII and HFD led to a significant increase in the expression of the CYP11B2 gene, a key gene involved in aldosterone synthesis than in the AII and ND groups (Fig. 5C). These results highlight the influence of dietary factors and AII treatment on adrenal gene expression, despite the absence of changes in plasma aldosterone levels.

## Discussion

Our findings demonstrate that oxLDL enhances AII-induced aldosterone production in an in vitro model of adrenocortical cells, likely via the AT1-LOX-1 receptor complex, leading to the activation of Gq signaling. This process is accompanied by calcium influx and upregulation of aldosterone synthesis-related mRNA, which is driven by Gq signaling activation [3]. Notably, using FlAsH-BRET biosensor analysis in CHO cells, we have previously demonstrated that simultaneous administration of oxLDL and Ang II induced a unique conformational change in AT1, thereby enhancing Gq signaling via the AT1-LOX-1 receptor complex [8, 14]. This phenomenon suggests that similar synergistic effects may occur in adrenal cells.

In the present study, we used IP1 accumulation and calcium influx as markers of Gq signaling activity in cells. Upon AII binding to the AT1, Gq protein activates phospholipase C-β (PLCβ), which hydrolyzes PIP₂ into diacylglycerol (DAG) and inositol trisphosphate (IP₃). IP₃ subsequently induces intracellular calcium release via its receptor on the endoplasmic reticulum [14]. While 10^−10^M AII alone induced transient calcium influx, the addition of oxLDL did not result in further increase in calcium mobilization, suggesting that calcium signaling had already reached a plateau (Fig. 2). In contrast, the same co-treatment significantly increased IP1 accumulation (Fig. 1), indicating that the Gq-PLC pathway activation was further enhanced. Because IP1 is a stable downstream metabolite of inositol trisphosphate and reflects cumulative PLC activity over time [15], it may serve as a more sensitive and integrated indicator of sustained Gq signaling than transient calcium flux, which is tightly regulated by homeostatic feedback mechanisms. Moreover, although calcium influx is a well-established activator of *CYP11B2* transcription, the upregulation of CYP11B2 gene expression observed under AII + oxLDL conditions occurred without a further increase in calcium influx, possibly suggesting that the transcriptional regulation of CYP11B2 gene may not be solely determined by peak calcium levels. While calcium influx remains essential, these results imply that prolonged or temporally integrated Gq signaling, as reflected by increased IP1 accumulation, may also contribute to the induction of CYP11B2 gene, potentially through repeated calcium transients or synergistic signaling downstream of the LOX-1–AT1 receptor interaction. Although calcium-dependent mechanisms remain central to the regulation of CYP11B2 gene, calcium-independent pathways, such as cAMP–PKA signaling, have also been proposed to regulate CYP11B2 expression independently of intracellular calcium mobilization [16, 17]. While such mechanisms were not the focus of the current study, they suggest the existence of additional upstream inputs that may act in parallel to or in coordination with Gq-PLC signaling. Collectively, these findings suggest that LOX-1–AT1 receptor interactions enhance Gq signaling in a manner that is not fully captured by calcium measurements alone. Whether the LOX-1–AT1 complexes contribute to regulation of CYP11B2 gene via additional calcium-independent mechanisms remains to be elucidated in the future.

In vivo experiments using two mouse models to examine acute and chronic stimulation with AII and oxLDL revealed no alterations in BP in response to oxLDL loading in AII-stimulated mice. However, short-term co-infusion of oxLDL with AII increased plasma aldosterone concentration without affecting aldosterone synthesis mRNA levels (Fig. 4C, D). Conversely, long-term treatment with a HFD and chronic AII infusion elevated aldosterone synthesis mRNA expression, particularly that of CYP11B2 gene, without altering plasma aldosterone levels (Fig. 5B, C), suggesting a dissociation between transcriptional activation and hormone secretion. This is consistent with previous findings showing that *CYP11B2* mRNA levels do not always correlate with aldosterone production in aldosterone-producing adenomas, likely due to post-transcriptional and enzymatic regulatory mechanisms [18]. These results imply that subpressor doses of AII in combination with HFD may activate adrenal steroidogenic pathways via LOX-1-dependent modulation of AT1 signaling, without necessarily inducing a marked increase in circulating aldosterone.

Additionally, LOX-1 deficiency attenuated the acute BP response to AII (Fig. 4B), whereas oxLDL alone did not elicit a comparable hypertensive effect. This discrepancy highlights the pivotal role of the LOX-1-AT1 receptor complex in modulating AII-induced BP responses. While AII activates AT1-Gq signaling, which leads to vasoconstriction and acute hypertension, oxLDL binding to LOX-1 preferentially engages AT1-associated Gi signaling and recruits β-arrestin, which facilitates internalization of the LOX-1–AT1 complex instead of activating the Gq-dependent vasoconstrictive pathway [7]. Consequently, oxLDL does not act as a direct hypertensive stimulus, rather it modulates AT1 signaling in a ligand-dependent manner.

Our previous work demonstrated that oxLDL alone activates ERK signaling via the LOX-1-AT1 complex in vascular endothelial cells through Gi/β-arrestin pathways without engaging Gq-dependent signaling [6, 7] More recently, we showed that co-treatment with AII and oxLDL induces a distinct AT1 receptor conformation that enhances Gq signaling in a LOX-1–dependent manner [8]. These findings support a model in which LOX-1 dynamically modulates AT1 receptor signaling depending on the ligand context: oxLDL alone biases signaling toward Gi/β-arrestin pathways, whereas the presence of AII facilitates enhanced Gq activation and downstream physiological responses through the LOX-1–AT1 complex. This mechanistic divergence explains why oxLDL alone fails to trigger aldosterone synthesis or BP elevation despite its ability to bind LOX-1. Although the relevance of AII-oxLDL crosstalk in vivo in the adrenal gland remains to be fully elucidated, our findings offer important insights into how these molecules may interact under specific physiological and pathological conditions.

Transcriptomic and proteomic datasets have confirmed that LOX-1 gene expression in the human adrenal gland is low but detectable (https://www.proteinatlas.org/ENSG00000173391-OLR1/tissue). As shown in Supplementary Fig., *LOX-1* and *AT1* (*AT1a* in mouse tissues) exhibited a relatively balanced expression profile in HASMCs and mouse aortas, whereas adrenal cells and tissues displayed predominant AT1 (or AT1a) gene expression with relatively low LOX-1 gene expression. Despite its low abundance, LOX-1 may still play a meaningful physiological role in the adrenal cortex through its physical and functional interactions with AT1, which is a key driver of aldosterone production and BP regulation [19]. Given the physical and functional interactions between AT1 and LOX-1 on cellular membranes, it is plausible that the role of LOX-1, despite its low expression, may be amplified by the abundant presence of AT1.

Furthermore, our earlier studies showed that oxLDL facilitates β-arrestin–mediated internalization of the AT1–LOX-1 complex and subsequent intracellular translocation of oxLDL itself [7]. This mechanism may be relevant to adrenocortical cells, which rely on intracellular cholesterol stored in lipid droplets as a substrate for aldosterone biosynthesis [20]. However, the mechanism by which oxLDL affects steroidogenesis in adrenocortical cells remains unclear. Therefore, investigating the role of LOX-1 in adrenal biology is critical to understand its broader implications.

This study underscores the potential therapeutic value of targeting the AT1-LOX-1 receptor complex or inhibiting Gq signaling to mitigate the adverse effects of oxLDL and AII on aldosterone production and cardiovascular health. Previous studies have shown increased LOX-1 expression under hypertensive conditions and demonstrated that LOX-1 KO mice exhibit attenuated responses of BP to AII infusion [12, 21]. These findings, together with our results, suggest that reducing the binding of oxLDL to LOX-1 may help lower BP. This aligns with evidence indicating that statins, commonly used for dyslipidemia, exert modest BP-lowering effects [4]. However, further research is required to resolve the absence of changes in BP observed in our models.

A significant limitation of our study is the discrepancy between the observed enhancement of AII-induced aldosterone production by oxLDL and lack of detectable differences in BP between treated and untreated mice. This may be attributed to the method employed to measure BP in our study. Specifically, the tailcuff method may lack sensitivity in detecting circadian variations or transient fluctuations that can be captured by telemetry [22]. Future studies that employ telemetry for continuous BP monitoring may provide a more nuanced understanding of the effects of oxLDL on BP regulation in the context of aldosterone production.

Additionally, histological and protein analyses of adrenal CYP11B2 were not performed. Such analyses could have clarified the discrepancies observed between CYP11B2 gene expression and plasma aldosterone concentrations in the two mouse models. Incorporating these methods in future studies may provide deeper insight into the molecular mechanisms governing aldosterone production in response to oxLDL and AII.

In conclusion, our study sheds light on the molecular mechanisms by which oxLDL enhances aldosterone production via the AT1-LOX-1 receptor complex and Gq signaling in adrenal cells. These findings highlight the interplay between dyslipidemia and RAAS, emphasizing their contribution to hypertension and cardiovascular diseases. This emphasizes the importance of using integrated therapeutic approaches to effectively manage these interconnected conditions.

## Supplementary information

Supplementary Table

Supplementary information

Supplementary Figure

## Data Availability

All relevant data are included in this paper. These datasets are available from the corresponding author upon reasonable requests.
