# Supplementary figures and images for: Oxidized LDL enhances Gq signaling and aldosterone production by angiotensin II via the AT1-LOX-1 receptor complex in adrenal cells
Source: Hypertens Res. 2025 Jun 18;48(9):2376–86. doi: 10.1038/s41440-025-02261-5 (PMC12411259; doi:10.1038/s41440-025-02261-5)

# Supplementary Fig.

**A**

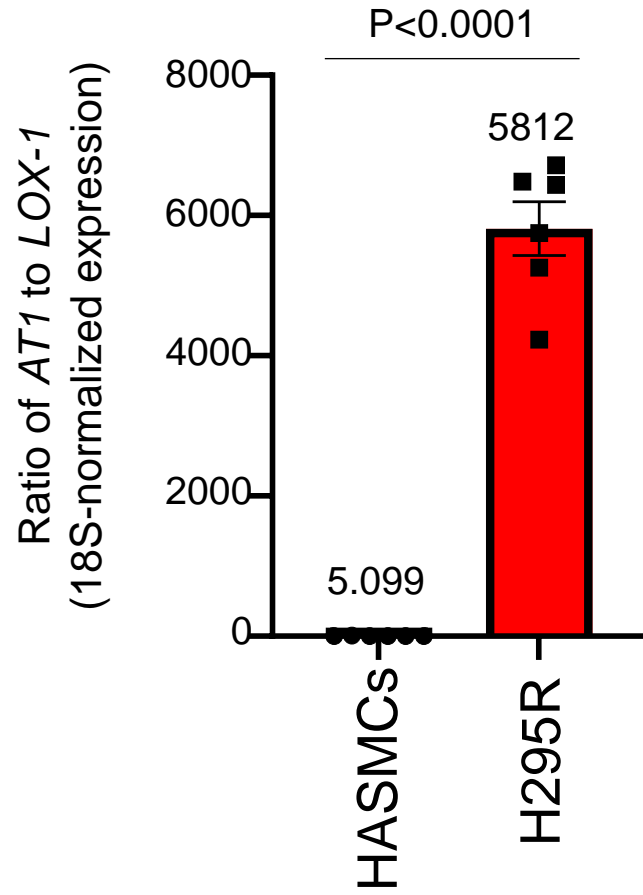

**B**

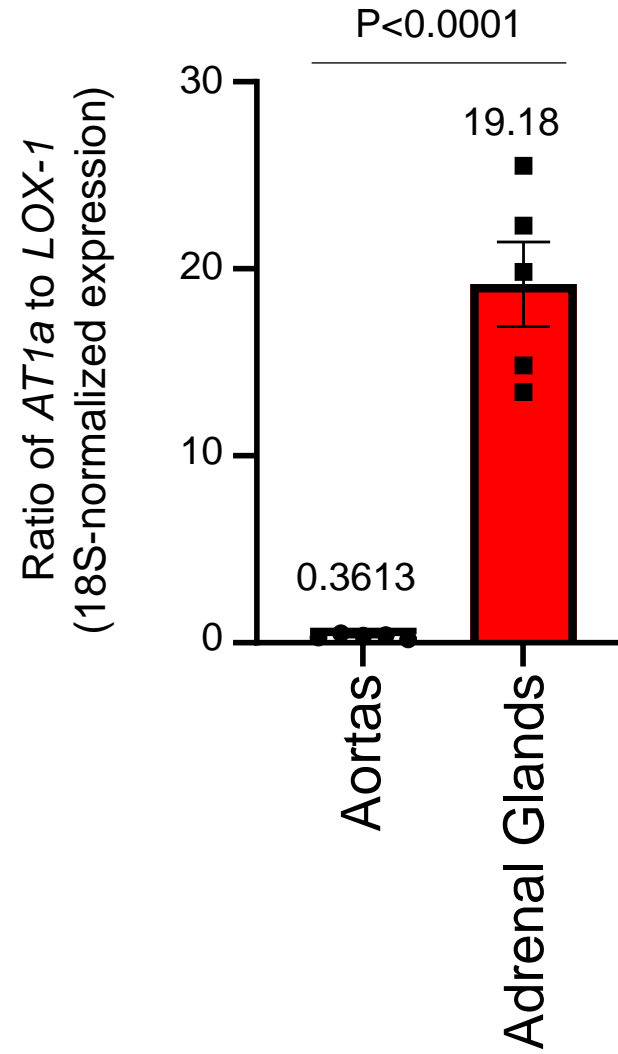

Supplement: Supplementary file 2 — Supplementary information [file 41440_2025_2261_MOESM2_ESM.pdf]
