# Supplementary material for: Oxidized LDL enhances Gq signaling and aldosterone production by angiotensin II via the AT1-LOX-1 receptor complex in adrenal cells
Source: Hypertens Res. 2025 Jun 18;48(9):2376–86. doi: 10.1038/s41440-025-02261-5 (PMC12411259; doi:10.1038/s41440-025-02261-5)
Supplement: Supplementary file 3 — Supplementary Figure [file 41440_2025_2261_MOESM3_ESM.docx]

**Supplementary Figure.** Differential expression balance of *LOX-1* and *AT1* (*AT1a* in mouse tissues) between vascular and adrenal cells and tissues.

(A) Expression ratio of *AT1* to *LOX-1* in HASMCs and H295R adrenocortical cells. (B) Expression ratio of *AT1a* to *LOX-1* in aortas and adrenal glands isolated from 9-week-old wild-type (WT) mice.

Expression ratios were calculated by dividing 18S-normalized *AT1* (or *AT1a*) mRNA levels by 18S-normalized LOX-1 mRNA levels, which were quantified using real-time qPCR.

Mean values for each group are indicated. Data are expressed as mean ± SEM. Statistical significance was assessed using Student’s *t*-test (n = 6 per group in A; n = 5 per group in B).
